# Effects of positive end-expiratory pressure on the predictability of fluid responsiveness in acute respiratory distress syndrome patients

**DOI:** 10.1038/s41598-021-89463-2

**Published:** 2021-05-13

**Authors:** Yen-Huey Chen, Ying-Ju Lai, Ching-Ying Huang, Hui-Ling Lin, Chung-Chi Huang

**Affiliations:** 1 Department of Respiratory Therapy, College of Medicine, Chang Gung University, Taoyuan, 33353 Taiwan; 2 Division of Pulmonary and Critical Care Medicine, Chang Gung Memorial Hospital, 5, Fu-Hsin St. Gweishan, Taoyuan, 33353 Taiwan; 3 Department of Respiratory Care, Chiayi Campus, Chang Gung University of Science and Technology, Chia-Yi, 61363 Taiwan; 4 Cardiovascular Division, Chang Gung Memorial Hospital Chang Gung University, Linkou, Tao-Yuan, 33353 Taiwan; 5 Department of Medical Imaging and Intervention, Chang Gung Memorial Hospital, Linkou, Tao-Yuan, 33353 Taiwan; 6 Department of Respiratory Therapy, Chang Gung Memorial Hospital, Linkou, Tao-Yuan, 33353 Taiwan

**Keywords:** Medical research, Respiratory distress syndrome

## Abstract

The prediction accuracy of pulse pressure variation (PPV) for fluid responsiveness was suggested to be unreliable in low tidal volume (VT) ventilation. However, high PEEP can cause ARDS patients relatively hypovolemic and more fluid responsive. We hypothesized that high PEEP 15 cmH_2_O can offset the disadvantage of low VT and improve the predictive performance of PPV. We prospectively enrolled 27 hypovolemic ARDS patients ventilated with low VT 6 ml/kg and three levels of PEEP (5, 10, 15 cmH_2_O) randomly. Each stage lasted for at least 5 min to allow for equilibration of hemodynamics and pulmonary mechanics. Then, fluid expansion was given with 500 ml hydroxyethyl starch (Voluven 130/70). The hemodynamics and PPV were automatically measured with a PiCCO2 monitor. The PPV values were significantly higher during PEEP15 than those during PEEP5 and PEEP10. PPV during PEEP15 precisely predicts fluid responsiveness with a cutoff value 8.8% and AUC (area under the ROC curve) of ROC (receiver operating characteristic curve) 0.847, higher than the AUC during PEEP5 (0.81) and PEEP10 (0.668). Normalizing PPV with driving pressure (PPV/Driving-P) increased the AUC of PPV to 0.875 during PEEP15. In conclusions, high PEEP 15 cmH_2_O can counteract the drawback of low VT and preserve the predicting accuracy of PPV in ARDS patients.

## Introduction

During inspiration with the support of mechanical ventilation, increased pleural and transpulmonary pressure reduces the right ventricular (RV) preload and increase the RV afterload, thus decreasing RV ejection. Because of the increase in the blood pulmonary transit duration, the inspiratory RV stroke volume decreases, which leads to a reduction in the expiratory left ventricular (LV) preload volume after a phase lag of two to three heart beats. Consequently, positive-pressure ventilation induces a cyclic inspiratory increase and an expiratory decrease in the LV stroke volume^1^. Michard F^1^ proposed that for hypovolemic patients on mechanical ventilation, respiratory variations in the pulse pressure and LV stroke volume are magnified because (1) the inspiratory transmission of pleural pressure into the right atrium (RA) increases when the RA is underfilled and compliant in terms of pressure and the venous system is collapsible by pleural pressure and (2) the inspiratory effect on the RV afterload is exaggerated, and both RV and LV are sensitive to preload change when they operate on the steeper portion of the Frank–Starling curve.

Recent systematic reviews^1–3^ have concluded that mechanical ventilation–induced dynamic preload indicators predict fluid responsiveness accurately in critically ill patients. Nearly all studies demonstrating high predictive accuracy of pulse pressure variation (PPV) have set the tidal volume (VT) to > 8–10 mL/kg.

Because of the markedly increased permeability of lungs after acute lung injury (ALI)^4^, acute respiratory distress syndrome (ARDS) is the most likely outcome of the deleterious effects of fluid overloading. Precisely predicting fluid responsiveness is crucial for avoiding hypovolemia-induced arterial hypotension and low cardiac output (CO) and for minimizing the risk of fluid overload–induced pulmonary edema exacerbation. To avoid volutrauma, the VT must be decreased to 6 mL/kg^5^, which is lower than the minimum requirement of 8 mL/kg proposed by De Backer^6^. With low VT ventilation, cyclic changes in pleural and intrathoracic pressure may not be sufficient to induce considerable cyclic variations in LV filling, which are required for identifying fluid responsiveness^7^. Therefore, the predictability of PPV for fluid responsiveness in ARDS patients has been demonstrated to be unreliable in studies applying a low VT^6,8–11^.

In addition to the low VT, adequate positive end-expiratory pressure (PEEP) for the reduction of ventilator-induced lung injury (VILI) from repetitive opening and closing of unstable lung units is another indispensable key requirement of the protective ventilatory strategy^12,13^. Recently, a meta-analysis^14^ of three multicenter controlled trials (Alveoli^15^, Lovs^16^, and Express^17^) concluded that higher levels of PEEP (15.3 versus 9.0 cmH2O) are associated with improved survival among ARDS patients.

PEEP affects the predictive performance of dynamic preload indicators through not only preload change but also complex heart–lung interactions^18^. Through the augmentation of both pleural and transpulmonary pressure, PEEP decreases venous return and increases the RV afterload, exacerbating preexisting hypovolemia. Therefore, high PEEP results in a leftward shift on the Frank–Starling curve to the steeper portion, making ARDS patients more hypovolemic and more fluid responsive^19^. High PEEPs have been demonstrated to increase PPV in ALI humans^19^ and increase RV and LV stroke volume variation (SVV) in animals^20^. Regarding the accurate prediction of fluid responsiveness, contrary to the findings of the aforementioned studies where all patients were ventilated with low PEEP, increased PEEP was used in ALI pig and septic ALI patients by da Silva Ramos et al.^21^ and Freitas et al.^22^, respectively, which revealed that PPV is a favorable predictor of hypovolemia. However, the effects of various PEEP levels on the predictive accuracy of fluid responsiveness of ARDS patients have seldom been reported. Therefore, this study investigated whether high PEEP can counteract the drawback of low VT (6 mL/kg) and whether PPV predicts fluid responsiveness more effectively when ventilated with high PEEP (15 cmH2O) or low PEEP (5 and 10 cmH2O) in the same ARDS patient.

## Materials and methods

### Patients

The Institutional Review Board (IRB) for Human Studies of Chang Gung Memorial Hospital approved the study protocols (IRB approval reference number 100-4473A3). Informed written consent was obtained from the patients’ nearest relatives. This study was performed in accordance with the Declaration of Helsinki and was registered in Clinical Trials.gov with the identifier NCT01716962. Patients who conformed to the Berlin definition of ARDS^23^ and were administered fluid resuscitation were enrolled. Their enrollment was based on the presence of at least one clinical sign of inadequate tissue perfusion, which included the following: (1) systolic blood pressure (BP) < 90 mm Hg or the need for vasopressors with norepinephrine; (2) urine output < 0.5 mL/kg/h for at least 2 h; (3) tachycardia (heart rate [HR] > 100/min); and (4) presence of skin mottling. Patients were excluded if they had cardiac arrhythmia or hemodynamic instability, defined as a variation in HR or BP by > 10% over a 15-min period, before the start of the procedure. Furthermore, patients with renal failure requiring renal replacement therapy were excluded.

### Measurement of CO, hemodynamics, PPV, and respiratory mechanics

Hemodynamic data, including HR, BP, cardiac index (CI), stroke volume index (SVI), PPV, and SVV, were downloaded directly and continuously at a frequency of once every 12 s from a bedside PiCCO2 hemodynamic monitoring system (version 3.1; Pulsion Medical Systems AG, Feldkirchen, Germany) in a spreadsheet format for subsequent calculations. The data of the last 2 min (11 sets of data) of each time interval were averaged to represent the hemodynamic data of that time interval. The PiCCO2 algorithm searches for the maximum and minimum pulse pressure in a 7.5-s window within the floating 30 s. PPV was calculated using the following equation: PPV = (PPmax − PPmin)/PPmean.

Respiratory mechanics, including the respiratory rate (RR), VT, plateau and mean airway pressure, PEEP and auto-PEEP levels, and respiratory system compliance (Crs), were derived beat-by-beat from the GALILEO GOLD ventilator (Hamilton Medical AG Via Nova Switzerland). The average of five successive data at the end of each time interval was used to represent the pulmonary mechanics of that setting interval. The GALILEO ventilator automatically measures Crs breath-by-breath by using a statistical technique called the least-squares fit method. This method is applied on a breath-by-breath basis, without the need for special inspiratory flow patterns and occlusion maneuvers, provided that the patient is relaxed.

### Mechanical ventilatory settings and study design

All patients were sedated with a continuous infusion of midazolam (F. Hoffmann-La Roche Ltd., Basle, Switzerland) and paralyzed with cisatracurium (F H Faulding & Co Limited, Victoria Australia) to abolish spontaneous triggering of mechanical ventilation. Patients were ventilated with the pressure-control mode; driving pressure was set to derive the VT to be approximately 6 mL/kg of the predicted body weight, and RR was set at 25 breaths/min. The mean PEEP level set before entry into the study was 14.3 (1.5) cmH2O. FiO2 was titrated to maintain SaO_2_ at > 90%. After the stabilization of hemodynamics at baseline, transpulmonary thermodilution cardiac output (CO) measurement was performed for calibrating pulse contour continuous CO. Following the calibration, patients received all the three levels of PEEP (5, 10, and 15 cmH2O) in a random order. Each PEEP level was maintained for at least 5 min to allow for the stabilization of hemodynamics, respiratory system mechanics, and arterial oxygenation. Arterial blood gas was drawn at the end of each PEEP setting. After all the three PEEP levels, the ventilator was returned to its prestudy settings. Then, volume expansions (VEs) were performed with the infusion of 500 mL hydroxyethyl starch (Voluven 130/0.4) at a rate of 10 mL/kg/h. Another transpulmonary thermodilution CO was then performed for calibration at the end of VEs.

According to the criteria used in previous studies^8,11,22^, patients were classified as VE responders or nonresponders depending on whether the VE-induced CI increase at the end of VE was ≥ 15% or < 15%. The rates of intravenous fluid and vasopressor infusion and the settings of mechanical ventilation were kept constant throughout the study period.

### Statistical analysis

All variables were indexed to the body surface area and are expressed as mean ± standard deviation (SD). The Kolmogorov–Smirnov test was used to verify the normality of the distribution of continuous variables. Significance differences of variables among the three PEEP levels were analyzed using the repeated measures analysis of variance (General Linear Model) or Friedman test followed by the Wilcoxon Signed Ranks test for nonparametric analysis. The differences in the various pre-VE values of responders and nonresponders were evaluated using the independent *t* test or Mann–Whitney *U* test. Spearman’s rank correlation was used to analyze the relationships of static lung compliance with PPV of the three PEEP levels before the fluid challenge. The prediction ability of the studied variables for positive fluid responsiveness after VE was tested using the receiver operating characteristic (ROC) curve. The cutoff value of the data is the maximum Youden’s index (J = sensitivity + specificity − 1). Sensitivity, specificity, and positive and negative predictive values are expressed as mean and 95% confidence intervals. All statistical analyses were conducted using SPSS for Windows (version 22, Chicago, IL, USA), and a *p* value of < 0.05 was considered statistically significant.

## Results

In total, 30 patients with early ARDS fulfilling the inclusion criteria were enrolled. The duration from ARDS diagnosis to the study procedures were within 2 days for all patients. Two patients were excluded because arrhythmia occurred during the study, and one patient was excluded because the VT was set too low (< 5 mL/kg). The main characteristics of the remaining 27 patients are summarized in Table 1. Among the ARDS patients enrolled in the study, 10, 13, and 14 had severe, moderate, and mild ARDS based on the Berlin definition.

**Table 1:** Patient characteristics at the time of study.

Age, years	61 (14.6) range:21–87 years
Gender, male:female	14:13
APACHE II score	24.7 (6.1)
**Main diagnosis**	
Pneumonia	18
Pulmonary alveolar hemorrhage	3
Viral pneumonia	3
Acute interstitial pneumonitis	3
Outcome of ICU stay, alive:dead	16:11
Time from diagnosis of ARDS to study	0–2 days
PaO_2_/FiO_2_ at diagnosis of ARDS mean (SD) mmHg)	124.4 (56.7); range 59.3–272
PEEP (cmH_2_O) set before entry into study	14.3 (1.5); range 10–16
**Case number of severe ARDS**	10 (37%)
Moderate ARDS	13 (48%)
Mild ARDS	4 (15%)
Norepinephrine infusion, yes:no	16 : 11

*APACHE II* acute physiology and chronic health evaluation II, *ICU* intensive care unit, *ARDS* acute respiratory distress syndrome.

The hemodynamics, pulmonary mechanics, blood gas data, and dynamic preload indicators of each time interval are listed in Table 2. Systolic and mean BP were significantly lower; the mean dynamic preload indicators, including SVV, PPV, PPV/Driving-P, and PPV/Crs, during PEEP 15 cmH2O were significantly higher than those during PEEP5 and PEEP10. Furthermore, PEEP15 had lower mean CI and SVI than did PEEP5. The mean Crs of PEEP15 was significantly lower, and the mean driving pressure of PEEP15 was significantly higher than that of PEEP10. VE significantly increased CI and SVI and decreased HR, SVV, PPV, PPV/Driving-P, and PPV/Crs. The responders’ SVV in all three PEEP levels and the PPV and PPV/Driving-P of PEEP5 and PEEP15 were significantly higher than those of nonresponders (Table 3). However, no significant differences were observed between responders and nonresponders for respiratory system mechanics in all the three PEEP levels.

**Table 2:** Hemodynamics, pulmonary mechanics, blood gas data, and preload indicators for each time interval.

Variable	PEEP 5	PEEP 10	PEEP 15	Pre Challenge	Post Challenge
HR (beats/min)	118 (22)	100 (31)	116 (22)	114 (21)	109 (21)*
SAP (mmHg)	136.9 (30.3)	134.8 (29.4)	123.2 (27.1)^a,b^	125.2 (25.1)	128.9 (21.5)
MAP (mmHg)	86.7 (20.9)	86.0 (19.9)	79.2 (20.1)^a,b^	81.5 (19.9)	85.2 (16)
CI (L/min/^.^m^2^)	3.88 (1.16)	3.78 (1.07)	3.66 (1.10)^a^	3.59 (1.09)	4.23 (1.31)*
SVI (mL/m^2^)	33.3 (8.8)	33.2 (8.2)	31.7 (8.6)^a^	31.6 (8.1)	38.5 (8.6)*
SVRI (dynes/sec/cm^5^/m^2^)	1555 (453)	1592 (507)	1479 (420)^b^	1549 (392)	1344 (535)*
Pplat (cmH_2_O)	20.4 (4.3)	24.32 (4.7)^c^	31.39 (5.3)^a,b^	30.36 (6.2)	29.96 (6.1)
Pmean (cmH_2_O)	10.39 (1.7)	15.13 (2.4)^c^	20.69 (2.1)^a,b^	19.6 (3.1)	19.7 (3.2)
Total PEEP (cm/H_2_O)	6.15 (1.1)	10.67 (0.8)^c^	15.54 (0.8)^a,b^	14.5 (1.5)	14.7 (1.6)
Crs (mL/cmH_2_O)	32.1 (14.8)	35.3 (15.0)	29.8 (14.1)^b^	29.9 (11.9)	30.5 (12.7)
Driving pressure (Pplat-total PEEP) (cm/H_2_O)	14.25 (4.3)	13.6 (4.9)	15.86 (5.4)^b^	15.9 (5.9)	15.3 (5.9)
SVV (%)	8.5 (5.4)	9.3 (5.3)	12.8 (7.2)^a,b^	11.6 (6.8)	7.8 (4.9)*
PPV (%)	5.9 (3.8)	6.2 (3.3)	9.7 (5.0)^a,b^	9.5 (5.4)	5.7 (3.3)*
PPV/driving-P (%)	0.46 (0.32)	0.52 (0.4)	0.68 (0.42)^a,b^	0.63 (0.38)	0.54 (0.69)*
PPV/Crs (%)	0.22 (0.18)	02.0 (0.12)	0.39 (0.27)^a,b^	0.38 (0.29)	0.24 (0.19)*
PaO_2_ mmHg	64.9 (17.3)	85.6 (31.1)^c^	89.2 (42.3)^a^		
PaO_2_/FiO_2_	109.2 (54.5)	138.7 (57)^c^	143.8 (63.8)^a^		
PaCO_2_ mmHg	60.8 (19.9)	60.9 (20.5)	62.4 (20)		
SaO_2_ (%)	86.0 (8.9)	92.5 (5.6)^c^	92.7 (5.0)^a^		

Values are mean (SD).

*HR* heart rate, *SAP* systolic arterial pressure, *MAP* mean arterial pressure, *CI* cardiac index, *SVI* stroke volume index, *SVRI* systemic vascular resistance index, *Pplat* plateau pressure, *Pmean* mean airway pressure, *Crs* respiratory system compliance, *PPV* pulse pressure variation, *SVV* stroke volume variation.

^a^Significantly different between PEEP 15 and PEEP 5.

^b^Significantly different between PEEP 15 and PEEP 10.

^c^Significantly different between PEEP 10 and PEEP 5.

*Significantly different between pre and post challenge.

**Table 3:** Hemodynamics, pulmonary mechanics, blood gas data, and preload indicators for responders and nonresponders at each positive end-expiratory pressure (PEEP) level.

Variable	PEEP 5		PEEP 10		PEEP 15	
	Responder (n = 16)	Nonresponders (n = 11)	Responder (n = 16)	Nonresponders (n = 11)	Responder (n = 16)	Nonresponders (n = 11)
HR (beats/min)	120 (22)	114 (22)	117 (23)	112 (24)	118 (22)	113 (22)
SAP (mmHg)	135 (32)	139 (29)	135 (34)	133 (25)	116 (25)	134 (28)
MAP (mm Hg)	84 (23)	90 (19)	85 (24)	86 (15)	73 (19)	88 (20)
CI (L/min/m^2^)	3.8 (1.15)	4.0 (1.22	3.8 (1.08	3.76 (1.09	3.51 (1.05)	3.88 (1.2)
SVI (mL/m^2^)	30.5 (7.4)	35.7 (9.7)	30.9 (7.3)	34.5 (9.7)	28.6 (7.5)	34.8 (9.5)
SVRI (dynes/s/cm^5^/m^2^)	1546 (470)	1571 (450)	1559 (517)	1612 (521)	1415 (411)	1571 (434)
PaO_2_ mmHg	60.0 (14.8)	71.8 (18.9)	80.8 (36.5)	92 (22)*	80.7 (46.7)	100.8 (34.3)*
PaO_2_/FiO_2_	91 (34.5)	134.2 (67.7)	117.1 (38.1)	168.3 (66.8)*	116.9 (48.9)	180.6 (65.3)*
PaCO_2_ mmHg	63.3 (22.7)	57.5 (15.8)	63.6 (23.4)	57.4 (16.3)	65 (22.1)	58.8 (17.3)
SaO_2_ (%)	83.1 (9.5)	89.6 (6.8)	90.6 (6.45)	95.1 (2.7)*	90.6 (5.3)	95.6 (2.7)*
Pplat (cmH_2_O)	21 (4.5)	18.9 (3.6)	24.7 (4.9)	23.5 (4.5)	32.2 (5.7)	29.5 (3.1)
Pmean (cmH_2_O)	10.8 (1.7)	9.7 (1.44)	15.4 (2.6)	14.7 (2.0)	21.1 (2.3)	20.1 (1.7)
Total PEEP (cm/H_2_O)	6.1 (1.2)	6.2 (0.8)	10.9 (1.4)	10.8 (0.6)	15.4 (0.7)	15.8 (0.9)
Crs (mL/cmH_2_O)	33.5 (16.7)	33.5 (15.8)	36.1 (15.8)	36.0 (16.0)	31.7 (14.8)	29.2 (14.5)
Driving pressure (Pplat-total PEEP) (cm/H_2_O)	14.3 (4.1)	14.2 (4.7)	13.0 (4.5)	14.1 (5.2)	15.5 (5.3)	16.4 (5.7)
SVV (%)	10.2 (5.4)	6.1 (3.8)*	11.2 (5.6)	6.5 (3.1)*	16.1 (7.5)	8.1) (3.0)*
PPV (%)	7.3 (4.1)	4.0 (2.4)*	7.0 (3.5)	5.1 (2.7)	12.1 (4.8)	6.2 (2.9)*
PPV/driving-P (%)	0.55 (0.32)	0.33 (0.27)*	0.64 (0.47)	0.6 (0.19)	0.9 (0.4)	0.36 (0.18)*
PPV/Crs (%)	0.27 (0.21)	0.13 (0.09)*	0.23 (0.13)	0.16 (0.11)	0.48 (0.29)	0.27 (0.20)

Values are mean (SD).

*HR* heart rate, *SAP* systolic arterial pressure, *MAP* mean arterial pressure, *CI* cardiac index, *SVI* stroke volume index, *SVRI* systemic vascular resistance index, *Pplat* plateau pressure, *Pmean* mean airway pressure, *Crs* respiratory system compliance, *PPV* pulse pressure variation, *SVV* stroke volume variation.

*Significantly different from its prior baseline value at 5% level.

**Figure 1:**
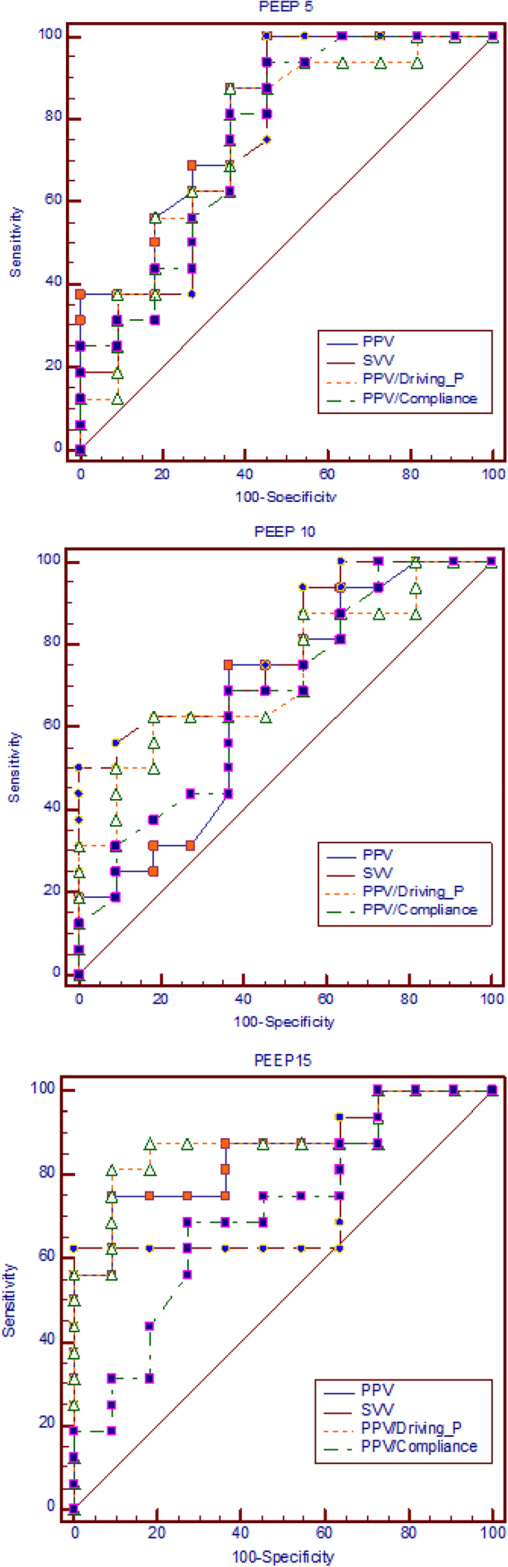
The receiver operating characteristic (ROC) curve of PPV, SVV, PPV/driving P, and PPV/compliance for the three PEEP levels.

For predictive performance, PPV during PEEP15 had the highest area under the curve (AUC) of the ROC curve (0.85) than those during PEEP5 (0.81) and PEEP10 (0.67). The cutoff value of PPV during PEEP15 was 8.8%. However, the cutoff value of PPV during PEEP5 (3.2%) was too low to be clinically significant. Indexing PPV with Crs did not significantly increase the AUC of all the three PEEP levels (Table 4). The ROC curves of PPV, SVV, PPV/Driving-P, and PPV/Compliance for each of the three PEEP levels are shown in Fig. 1.

**Table 4:** Predictive performance of fluid responsiveness for PPV, SVV, PPV/driving-P, and PPV/Crs among the three PEEP levels.

	PPV	SVV	PPV/driving-P	PPV/Crs
**PEEP 5**				
AUC	0.81 (0.61–0.93)	0.75 (0.55–0.90)	0.75 (0.55–0.90)	0.76 (0.55–0.90)
Cutoff	3.2	4.5	0.26	0.1
Sensitivity (%)	100 (79.4–100)	100 (79.4–100.0)	87.5 (61.7–98.4)	93.6 (69.8–99.8)
Specificity (%)	54.6 (23.4–83.3)	54.6 (23.4–83.3)	63.6 (30.8–89.1)	54.6 (23.4–83.3)
**PEEP 10**				
AUC	0.67 (0.46–0.84)	0.79 (0.59–0.92)	0.72 (0.52–0.88)	0.67 (0.47–0.84)
Cutoff	4.6	10.4	0.42	0.15
Sensitivity (%)	75 (47.6–92.7)	50 (24.7–75.3)	62.5 (35.4–84.8)	68.8 (41.3–89.0)
Specificity (%)	63.6 (30.8–89.1)	100 (71.5–100)	81.8 (48.2–97.7)	63.6 (30.8–89.1)
**PEEP 15**				
AUC	0.85 (0.66–0.96)	0.76 (0.55–0.90)	0.88 (0.69–0.97)	0.71 (0.50–0.86)
Cutoff	8.8	11.5	0.48	0.32
Sensitivity (%)	75 (47.6–92.7)	62.5 (35.4–84.8)	81.3 (54.5–96.0)	68.8 (41.3–89.0)
Specificity (%)	90.9 (58.7–99.8)	100 (71.5–100)	90.9 (58.7–99.8)	72.7 (39.0–94.0)

*AUC* area under the receiver operating characteristic curve, *PPV* pulse pressure variation, *SVV* stroke volume variation, *Crs* respiratory system compliance.

Normalizing PPV based on Driving-P further increased the AUC from 0.85 for PPV to 0.88 for PPV/Driving-P during PEEP15. However, the prediction performance remained poor for PEEP10 (AUC 0.67 versus 0.72) and was even poorer for PEEP5 (AUC 0.81versus 0.75) (Table 4).

No significant correlation was observed between PPV and Crs for all the three PEEP levels irrespective of the total cases (*p* value for PEEP5: 0.77; PEEP10: 0.99; and PEEP15: 0.32) or fluid responders (*p* value: 0.83, 0.97, and 0.32, respectively). The AUCs of PPV of Crs ≤ 30 versus Crs > 30 for PEEP5, PEEP10, and PEEP15 were 0.49 versus 0.55, 0.56 versus 0.70, and 0.49 versus 0.54, respectively.

## Discussion

The main findings of our study are as follows: (1) high PEEP of 15 cmH2O considerably decreased hemodynamics but increased the dynamic preload indicators of PPV, SVV, and PPV/Driving-P compared with those of PEEP5 and PEEP10; (2) Despite low VT, automatically measured PPV during PEEP15 had high predictive accuracy for fluid responsiveness, with the cutoff value of 8.8% and AUC of 0.85, which are higher than the AUC of PPV during PEEP5 (0.81) and PEEP10 (0.67); (3) correcting PPV with driving pressure further improved the prediction performance of PPV of PEEP15. Our study entirely focused on ARDS patients and investigated the effects of various PEEP levels on PPV predictability for fluid responsiveness. Furthermore, three PEEP levels were maintained in the same ARDS patients rather than analyzing various PEEPs in different patient groups who were already ventilated.

Up to now, the predictive accuracy of PPV for fluid responsiveness in ARDS patients ventilated with low VT has been controversial and low. The main discrepancy causing conflicting results is PEEP level setting. With low VT along with low PEEP, several animal^9^ and human studies have demonstrated that PPV is a poor indicator of fluid responsiveness. In the ARDS subgroup in De Backer’s study with a VT of < 8 mL/kg and PEEP of 11 cmH2O, the reported AUC of ROC curve was 0.71^6^. Similarly, low AUC (0.64) was also found in the low VT group (6.6 mL/kg) (20/42 patients had ARDS) of Vallee’s study with PEEP set only at 6 (4–8) cmH2O [8]. Moreover, Lakhal et al.^10^ indicated that the performance of PPV was poor (AUC 0.75) in 65 people with ARDS ventilated with low VT [6.5 (1.4) mL/kg] and low PEEP [8.5 (3.2) cmH2O]. Recently, Monnet et al. reported a low AUC of 0.69 in a low-compliance ARDS group^11^, with PEEP and VT set at 7 cmH2O and 7.1 mL/kg, respectively. Contrary to the aforementioned studies involving low PEEP, Michard et al.^19^ demonstrated that PPV increased from 9% ± 7% to 16% ± 13% in ALI patients ventilated with VTs of 8–12 mL/kg when the PEEP increased from 0 to 10 cmH2O. Furthermore, both Kubitz et al.^20^ and Renner et al.^25^ showed increased PPV values with high PEEPs in anesthetized pigs. The excellent predictive performance of PPV in patients ventilated with high PEEP were demonstrated in septic ALI patients by Freitas et al. [median PEEP 10 (10.0–13.5) cmH_2_O, AUC 0.91]^22^ and in ALI pigs by da Silva Ramos et al. (PEEP 13 cmH2O, AUC 0.88)^21^. Our data support the aforementioned inferences and show that PPV during PEEP15 had higher AUC (0.85) than that during PEEP10 (0.67) and PEEP5 (0.81) (Table 4 and Fig. 1). Furthermore, the mean PPV value during PEEP15 (9.7%) was considerably higher than those during PEEP10 (6.2%) and PEEP5 (5.9%) (Table 2). Moreover, the low cutoff values of PEEP5 (3.2%) and PEEP10 (4.6%) are vulnerable to the influence of small measurement errors and signal noise, making its clinical use for differentiating volume responders and nonresponders unreliable. Using our data, we confirmed our hypothesis that high PEEP can counteract the adverse effects of small variations in pleural and transpulmonary pressure from a small VT for predicting fluid responsiveness.

Respiratory changes in pleural and intrathoracic pressure depend on the alveolar pressure variation and lung compliance, influencing the degree of airway pressure transmission to pleural spaces. False-negative results may occur even in patients with preload responsiveness if the change in intrathoracic pressure from low VT is too small to produce a sufficient preload change. Normalizing PPV by using driving pressure attenuates the false-positive or false-negative rate of PPV^8^. However, the reliability remained poor for patients with a VT of < 8 mL/kg and low PEEP of 6 cmH2O (AUC for PPV: 0.63 and PPV/Driving-P: 0.72) in Vallee’s study^8^. Furthermore, Lakhal et al. reported that in ARDS patients with low VT (6.5 mL/kg) and low PEEP (8.5 cmH2O) and PPV/Driving-P (AUC 0.72) did not improve the AUC of PPV (0.75)^10^. This negative performance is because pressure transmission from airway to pleura cannot be reliably estimated from the difference between plateau and PEEP^28^. In our data, the PPV/Driving-P during PEEP15 was significantly higher than during PEEP10 and PEEP5 (Table 2). Similar to the results of both Vallee^8^ and Lakhal^10^, PPV/Driving-P did not improve the predictive accuracy of PPV during PEEP5 (AUC 0.81 for PPV versus 0.75 for PPV/Driving-P) and PEEP10 (AUC 0.67 versus 0.72) in the current study. However, the predictive performance of PPV/Driving-P improved compared with PPV during PEEP15 (AUC 0.88 versus 0.85) (Table 4).

With a decrease in Crs, the transmission of Paw to pleural space decreases, potentially attenuating the effect of increased airway pressure on RA pressure and pressure gradient for venous return^27–29^. Teboul et al.^30^ proposed that low VT and low lung compliance account for the poor ability of PPV for predicting fluid responsiveness in ARDS. Recently, Monnet et al.^12^ demonstrated that the prediction ability of PPV is inversely related to Crs. If Crs was < 30 mL/cmH2O, then the PPV became less accurate for predicting fluid responsiveness. On the contrary, Venus et al.^31^ demonstrated that airway pressure transmission to pleural space reduced in ALI. However, with a constant VT, pleural pressure changes remained unaltered by changes in lung compliance. Scharf and Ingram^32^ and Romand et al.^29^ have proposed that the primary determinant of changes in pleural or intrathoracic pressure is the amount of lung inflation and not a specific change in compliance. Lakhal et al.^10^ demonstrated that PPV performance in patients with Crs less and more than its median value is similar, suggesting that this respiratory parameter fails to identify patients with higher PPV performance. Moreover, we did not find a considerable correlation between PPV and Crs in the total number of cases or in fluid responders for all three PEEP levels. The predictive accuracy of subgroups with Crs > 30 mL/H2O was not higher than that of subgroups with Crs < 30 mL/H2O. Furthermore, unlike PPV/Driving-P, adjusting PPV based on Crs even worsened the predictive performance of PPV in all three PEEP levels (Table 4). Our study design is different from that of Monnet et al.^11^, in that we fixed the VT during ventilation with different PEEP levels. When determining the hemodynamic effects of PEEP, Luecke T^18^ suggested that the crucial question is to what extent will PEEP change the total lung volume and intrathoracic pressure. Therefore, the constant VT during various PEEP levels in our study might account for the Crs not affecting the accuracy of PPV for predicting fluid responsiveness.

The sampling duration and manual measurement of PPV also influence the precision and magnitude of PPV. Kim and Pinsky^33^ demonstrated that PPV increased progressively with the increasing sampling duration up to but not exceeding five breaths. As shown in the Methods section, the PiCCO2 monitor determines the maximum and minimum pulse pressure and stoke volume in an interval of 7.5 s; the average of the four 7.5-s intervals within a continuous sliding time widow of 30 s was used to calculate PPV and SVV. With an RR of 25 breaths/min, the sampling duration of 7.5 s comprises more than three breaths. Furthermore, the last 2 min of data were averaged to represent the hemodynamic data of that time interval to minimize artifact and noise of measurement in our study. During the manual estimation of PPV, small measurement errors in pressure frequently occur; therefore, Vallee et al.^8^ suggested that artifacts and noise in the calculated PPV^22^ are responsible for the low predictive precision of PPV. Some studies that calculated PPV manually have found the predictive performance of PPV to be unsatisfactory^6,10^. Unlike previous studies in which COs were measured through echocardiography or the thermodilution method, which are prone to interobserver bias and measurement error, the hemodynamic data and PPV in our study were automatically calculated by the same PiCCO2 monitor. In addition to a high PEEP, automatic measurement and the long sampling duration of our hemodynamic data and PPV are the other possible explanations for the high AUC of PPVs observed in our study.

However, our study did have the limitation that the PA pressure was not obtained from the PiCCO2 monitor, and no echocardiography was performed to determine whether false-positive results were caused by acute cor pulmonale resulting from RV dysfunction. However, Vieillard-Baron et al.^34^ suggested that the occurrence of acute cor pulmonale, as a ARDS complication, is progressive and usually requires at least 48 h of respiratory support. Furthermore, using echocardiography, they found that the incidence of acute cor pulmonale in ARDS declined from 61% during ventilation with high VT (13 mL/kg) to 25% under protective ventilation (6–9 mL/kg). Nearly all of our ARDS patients were enrolled within 2 days of ARDS diagnosis and were ventilated with low VT (6 mL/kg). Therefore, the influence of acute cor pulmonale on the predictive accuracy of PPV may not be as serious in our study. Table 2 showed that PEEP15 resulted in a considerable decrease in Crs, MAP, and CI as compared with low PEEP. Thus, high PEEP may contribute to lung overinflation and potentially promote VILI. For hemodynamically unstable patients, increasing PEEP to 15 cmH2O, although cautiously, should be limited in time to limit the adverse effects on the patient’s hemodynamic state.

In conclusion, a high PEEP of 15 cmH2O considerably decreases the hemodynamics and increases the PPV. Ventilation with a high PEEP of 15 cmH2O can offset the disadvantage of low VT and preserve the high predictability of automatically calculated PPV for fluid responsiveness of ARDS patients. Normalizing PPV with driving pressure can further improve PPV predictability during a PEEP of 15 cmH2O.
